# Improving CRISPR/Cas9 mutagenesis efficiency by delaying the early development of zebrafish embryos

**DOI:** 10.1038/s41598-020-77677-9

**Published:** 2020-12-03

**Authors:** M. Terzioglu, A. Saralahti, H. Piippo, M. Rämet, J.-O. Andressoo

**Affiliations:** 1 Department of Pharmacology, Faculty of Medicine and Helsinki Institute of Life Science, University of Helsinki, Helsinki, Finland; 2 BioMediTech, Faculty of Medicine and Health Technology, Tampere University, Tampere, Finland; 3 Department of Neurobiology, Care Sciences and Society, Karolinska Institutet, Solna, Sweden

**Keywords:** CRISPR-Cas9 genome editing, Zebrafish

## Abstract

CRISPR/Cas9 driven mutagenesis in zygotes is a popular tool for introducing targeted mutations in model organisms. Compared to mouse, mutagenesis in zebrafish is relatively inefficient and results in somatic mosaicism most likely due to a short single-cell stage of about 40 min. Here we explored two options to improve CRISPR/Cas9 mutagenesis in zebrafish—extending the single-cell stage and defining conditions for carrying out mutagenesis in oocytes prior to in vitro fertilization. Previous work has shown that ovarian fluid from North American salmon species (coho and chinook salmon) prolong oocyte survival ex vivo so that they are viable for hours instead of dying within minutes if left untreated. We found that commonly farmed rainbow trout (*Oncorhynchus mykiss*) ovarian fluid (RTOF) has similar effect on zebrafish oocyte viability. In order to prolong single-cell stage, we incubated zebrafish zygotes in hydrogen sulfide (H_2_S) and RTOF but failed to see any effect. However, the reduction of temperature from standard 28 to 12 °C postponed the first cell division by about an hour. In addition, the reduction in temperature was associated with increased CRISPR/Cas9 mutagenesis rate. These results suggest that the easily applicable reduction in temperature facilitates CRISPR/Cas9 mutagenesis in zebrafish.

## Introduction

Understanding of human development and disease requires information obtained from genetic model organisms. Besides many other advantages, zebrafish (*Danio rerio*) offers a unique model with externally fertilized eggs and synchronously developing transparent embryos^1^. Importantly, 70% of human genes are related to genes found in zebrafish and 85% of disease associated human genes have a zebrafish orthologue, making zebrafish a relevant organism for studying human diseases^2^.

Genetic studies in zebrafish have traditionally been carried out with the Morpholino-oligonucleotide (MO) knockdown method which, however, has received criticism due to the disadvantageous off-target effects^3^. The Clustered Regularly Interspaced Short Palindromic Repeats (CRISPR)—CRISPR*-*associated (Cas9) enzyme-based knockout technology, on the other hand, provides a powerful tool with easy design and relatively low mutagenesis rate at potential off-target sites^4,5^. To achieve targeted mutations in the zebrafish genome with CRISPR/Cas9 method, a Cas9 endonuclease and the target specific single guide RNA (sgRNA) are microinjected into fertilized eggs at early 1-cell stage^4,5^. This causes a double-strand break and produces indels by the subsequent actions of Cas9 endonuclease and cells own repair mechanisms. In most cases, however, the injection gives rise to mosaic animals with multiple, independent genetic changes which complicates the phenotypic outcome and thus further genotype–phenotype correlation analyses in the F0 generation^6,7^. To overcome these difficulties, phenotypic analyses are usually conducted in the homozygous fish of F2 or further generations. However, genotyping and breeding of multiple generations is time consuming, expensive and laborious.

Several approaches have been tested to increase the mutagenesis efficiency and the germline transmission of CRISPR/Cas9 method, including the use of Cas9 protein instead of mRNA or plasmids, accelerating Cas9 degradation process in order to avoid persistent mutagenesis and, most importantly, introducing genetic changes as early as possible in relation to DNA replication and cell division in mutated embryo (reviewed e.g. in Ref.^8^. In mice, Hashimoto et al.^9^ were able to generate biallelic, non-mosaic mutants by delivering CRISPR components to in vitro fertilized oocytes by electroporation providing proof for the importance of early mutagenesis. In addition, Xie et al.^10^, were able to increase the mutagenesis rate in zebrafish embryos by introducing Cas9 and sgRNA into oocytes instead of fertilized eggs. In conclusion, to produce a single, ubiquitous mutation in an embryo, introduction of the genetic change should ideally occur before the first cell division. However, the first cell division in a zebrafish zygote takes place in a very short time post fertilization (in around 40 min), giving Cas9 and the repair mechanisms relatively little time to achieve the mutation. In contrast, in mice the first cell division occurs several hours after fertilization providing more time for the mutagenesis to occur.

Here we hypothesized that the mutagenesis efficiency of CRISPR/Cas9 method could be improved at least by two ways, by introducing mutations into zebrafish oocytes prior to in vitro fertilization or by extending the one-cell stage in zebrafish zygotes. Oocyte manipulation requires an appropriate medium to preserve the viability of oocytes during the injection and the following incubation, as when in contact with water, oocytes die within minutes. Here we report that, as previously reported ovarian fluids of coho salmon (*Oncorhynchus kisutch*) and chinook salmon (*Oncorhynchus tshawytscha*)^11,12^, the ovarian fluid from commonly farmed rainbow trout (*Oncorhynchus mykiss*) preserves the viability of isolated zebrafish oocytes for at least 4 h.

To delay the first cell division in zebrafish zygotes, we tested three treatments—incubation of zygotes in RTOF or hydrogen sulfide (H_2_S) or reduction of embryonic temperature. Using time-lapse imaging we found that by incubating zygotes at 12 °C, one-cell stage is extended from 40 min to about 70–100 min without causing abnormal development. Furthermore, by using previously tested and characterized sgRNAs, we found that incubation of embryos at 12 °C for 30–60 min after Cas9/sgRNA injection increases the mutagenesis efficiency. These results suggest that the reduction of temperature from the normal 28 to 12 °C improves Cas9 driven mutagenesis in zebrafish without causing side-effects.

## Results

### Analysis of the effect of the rainbow trout ovarian fluid on the viability of zebrafish oocytes

**Figure 1:**
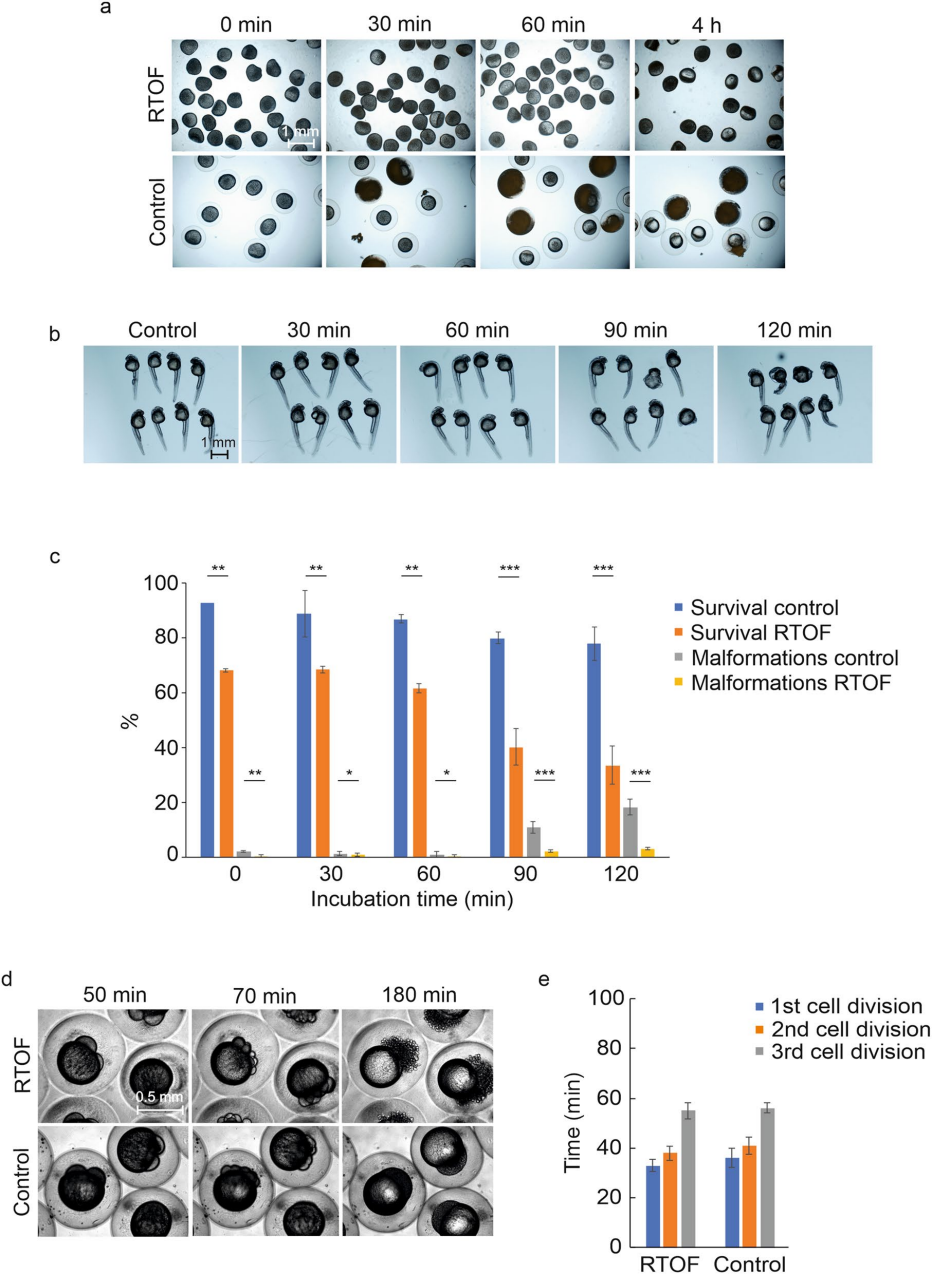
The effect of RTOF treatment on the oocyte viability and the early development of zebrafish embryos. (**a**) RTOF preserves zebrafish oocyte viability for at least 4 h. Oocytes were harvested by squeezing the abdomen of a competent female and incubated in RTOF or control solution (E3 water) for 4 h. Oocytes were imaged at 0, 30 and 60 min, and at 4 h. Oocytes kept in RTOF were alive and had attached chorions as a sign of preserved competence for fertilization, throughout the experiment. In contrast, control oocytes exhibited expanded chorions immediately after collection and started dying at 30 min post collection. (**b**,**c**) 30–60 min treatment with RTOF causes minor defects in the morphology and the survival of zebrafish embryos, while 90–120 min treatment causes substantially increased malformation and mortality. Fertilized zebrafish eggs were incubated in RTOF for 30, 60, 90 and 120 min after which they were moved to E3 water. The survival and well-being of the embryos was followed for 48 h and compared to E3 treated group. n(Control) = 348, n(RTOF 30 min) = 296, n(RTOF 60 min) = 202, n(RTOF 90 min) = 196, n(RTOF 120 min) = 240. (**d**) The comparative developmental stages of the control (E3 treated) and the RTOF treated embryos at indicated time points post fertilization. Newly fertilized eggs were kept in RTOF and their development was followed by time-lapse imaging. (**e**) The times for the first, the second and the third cell division in RTOF treated and control (E3 treated) embryos. Error bars represent SD, *p < 0.05, **p < 0.01, ***p < 0.001.

Genetic manipulation of zebrafish oocytes rather than zygotes requires an appropriate medium to preserve the viability and fertilizability of the isolated oocytes throughout the injection and incubation period as without a proper preservation medium, the oocytes lose the competence for fertilization and die within minutes^11,12^. Because neither coho or chinook salmon ovarian fluids are not commercially available, we approached fish farm producing rainbow trout, a commonly farmed species in Northern Europe and obtained ovarian fluid. We found that rainbow trout ovarian fluid (RTOF), preserves the viability of zebrafish oocytes for at least 4 h (Fig. 1a). We also assessed the competence of the oocytes for in vitro fertilization by morphological observation, namely, the attachment of the chorion on the yolk which has previously been noted to be critical for the success of in vitro fertilization^12^. Oocytes stored in RTOF stayed alive and had attached chorions throughout the experiment (up to 4 h), while control oocytes exhibited expanded chorions within seconds of contact with E3 water and started dying 30 min post exposure (Fig. 1a).

We also examined the competence for fertilization. To this end, we in vitro fertilized oocytes stored in RTOF for 0, 1 or 2 h and monitored their survival. While about 30% of the control oocytes fertilized immediately after harvest survived until 24 hpf (hours post fertilization), survival percentages of 9, 11, and 3% were obtained for the oocytes stored in RTOF for 0, 1, and 2 h, respectively (Supplementary Fig. S1).

### Analysis of the effect of RTOF on the timing of the first cell division in zebrafish embryo

Next, we tested whether incubating zebrafish zygotes in RTOF delays the onset of first cell division compared to the zygotes kept in E3 water. We first tested the effect of different incubation times, 0, 30, 60, 90 or 120 min, on the survival and well-being of the embryos during the first two days of development. Embryos were incubated in RTOF in 28 °C for the desired times after which they were moved to E3 medium and imaged at 48 hpf. Noteworthy, 30 and 60 min RTOF treatment of fertilized eggs caused slightly increased mortality (32% for 30 min, 38% for 60 min) and comparable levels of malformed embryos (1.4% for 30 min, 1% for 60 min) compared to the E3 treated embryos (mortality 11%, malformed embryos 1%) (Fig. 1b,c) (p < 0.01). However, embryos treated with RTOF for 90 and 120 min exhibited substantially increased levels of both (mortality: 60% for 90 min, 66% for 120 min; malformation: 11% for 90 min, 18% for 120 min) (Fig. 1b,c) (p < 0.001). To determine the effect of RTOF on the developmental rate, we performed time-lapse imaging of newly fertilized zebrafish eggs. The eggs were kept in RTOF throughout the imaging and the time for the first, the second and the third cell division was determined and compared to the control embryos kept in E3 medium. Overall, we did not observe significant difference in the timing of cell divisions between RTOF treated embryos and controls. Instead, we observed comparable times for the first (33 min for RTOF, 35 min for E3), the second (37 min for RTOF, 41 min for E3) and the third (54 min for RTOF, 56 min for E3) cell division in both groups, with a minor delay of 3 min in RTOF treated embryos (p < 0.05) (Fig. 1d,e, Supplementary Video S1). Noteworthy, embryos kept in RTOF showed remarkable defects in development from the 8-cell stage forward further illustrating the infeasibility of this approach (Fig. 1d).

### Analysis of the effect of hydrogen sulfide (H_2_S) induced “suspended animation-like” condition on the timing of the first cell divisions in zebrafish

**Figure 2:**
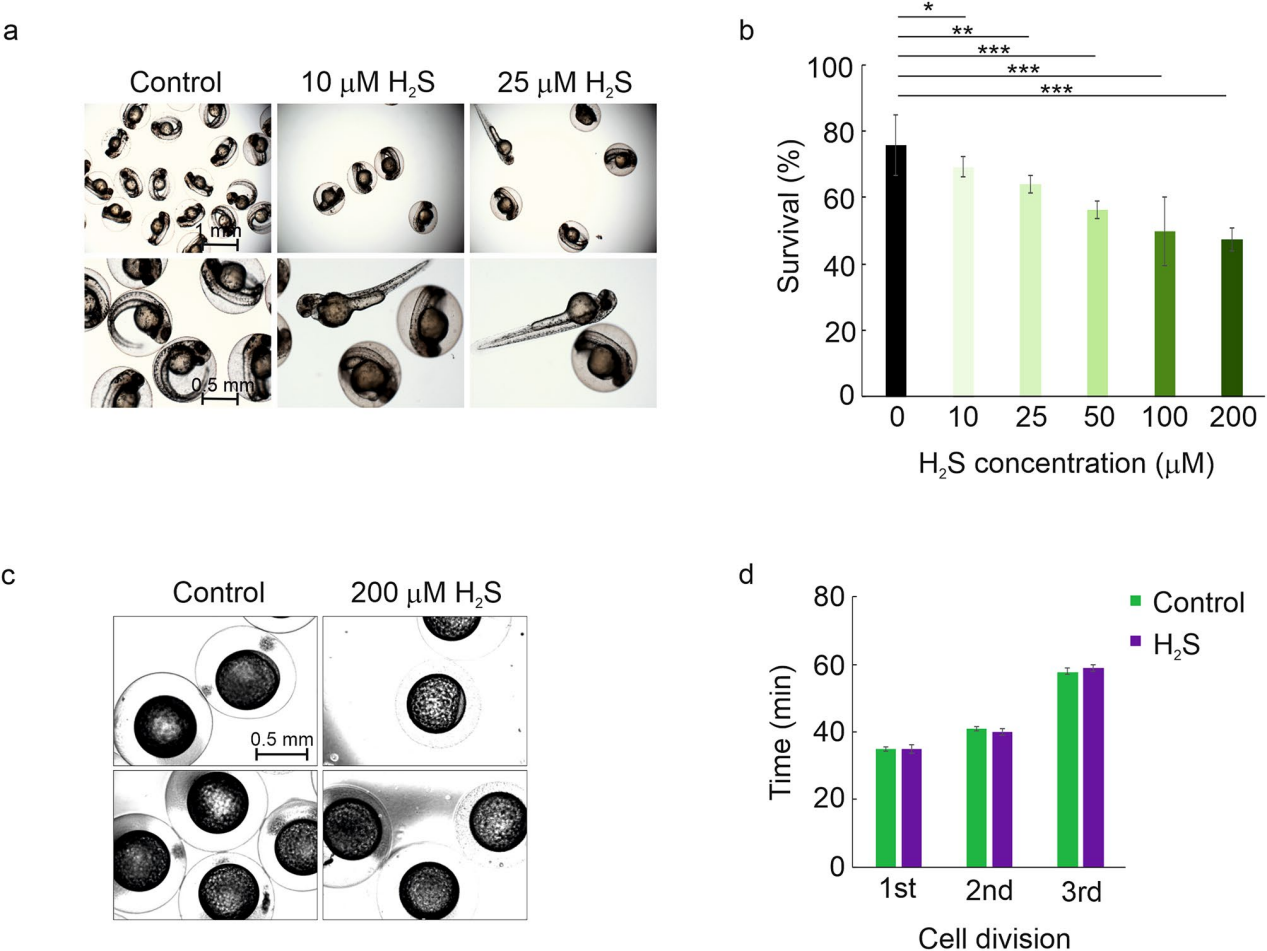
The effect of H_2_S treatment on the early development of zebrafish embryos. (**a,b**) H_2_S concentrations of 10–25 µM cause no changes in the morphology and minor decrease in the survival of zebrafish embryos, while the concentrations of 50–200 µM cause substantial increase in both the rate of malformation and mortality. Fertilized eggs were incubated in E3 water supplemented with 10–200 µM H_2_S for 48 h. The survival and well-being of the embryos was followed for 48 h and compared to E3 treated group (control). n(Control) = 397, n(10 µM) = 358, n(25 µM) = 310, n(50 µM) = 130, n(100 µM) = 182, n(200 µM) = 208. (**c**) Comparative developmental stages of control (E3 treated) and H_2_S treated embryos. Newly fertilized eggs were kept in E3 water with 200 µM H_2_S and the development was followed by time-lapse imaging. Two representative images are shown for both treatments (control images on the left, H_2_S treatment images on the right). (**d**) The times for the first, the second and the third cell division in H_2_S treated and control (E3 treated) embryos. Error bars represent SD, *p < 0.05, **p < 0.01, ***p < 0.001.

Next, we tested whether hydrogen sulfide affects the rate of zebrafish development. We incubated newly fertilized eggs in serial concentrations (from 10 μM to 200 μM) of H_2_S and imaged them 48 hpf. The moderate doses (10–25 µM) of H_2_S did not have a noticeable effect on the morphology of the embryos during the 48-h follow-up period (Fig. 2a). Similarly, with the concentrations from 10 to 25 μM embryo survival was only slightly affected, 69–64%, compared to the control group (76%) (Fig. 2b). In contrast, H_2_S treatment with concentrations from 50 to 200 μM increased the overall mortality of the treated embryos within the first two days of development, resulting in the survival rates ranging from 47 to 56% while survival percent of 76% was observed in control embryos treated with E3 water (Fig. 2b). Next, we analyzed if H_2_S delays the early development of the embryos. Similar to RTOF experiments, we performed time-lapse imaging of newly fertilized zebrafish eggs in 200 μM hydrogen sulfide at 28 °C for 6 h. However, time-lapse experiments showed no developmental delay upon H_2_S treatment, where the times for the first, the second and the third cell division were 35, 40 and 59 min, respectively, compared to the E3 water treated control embryos for which the corresponding times were 35, 41 and 58 min (Fig. 2c,d, Supplementary Video S1).

### Analysis of the effect of reduced temperature on the timing of first cell divisions in zebrafish

**Figure 3:**
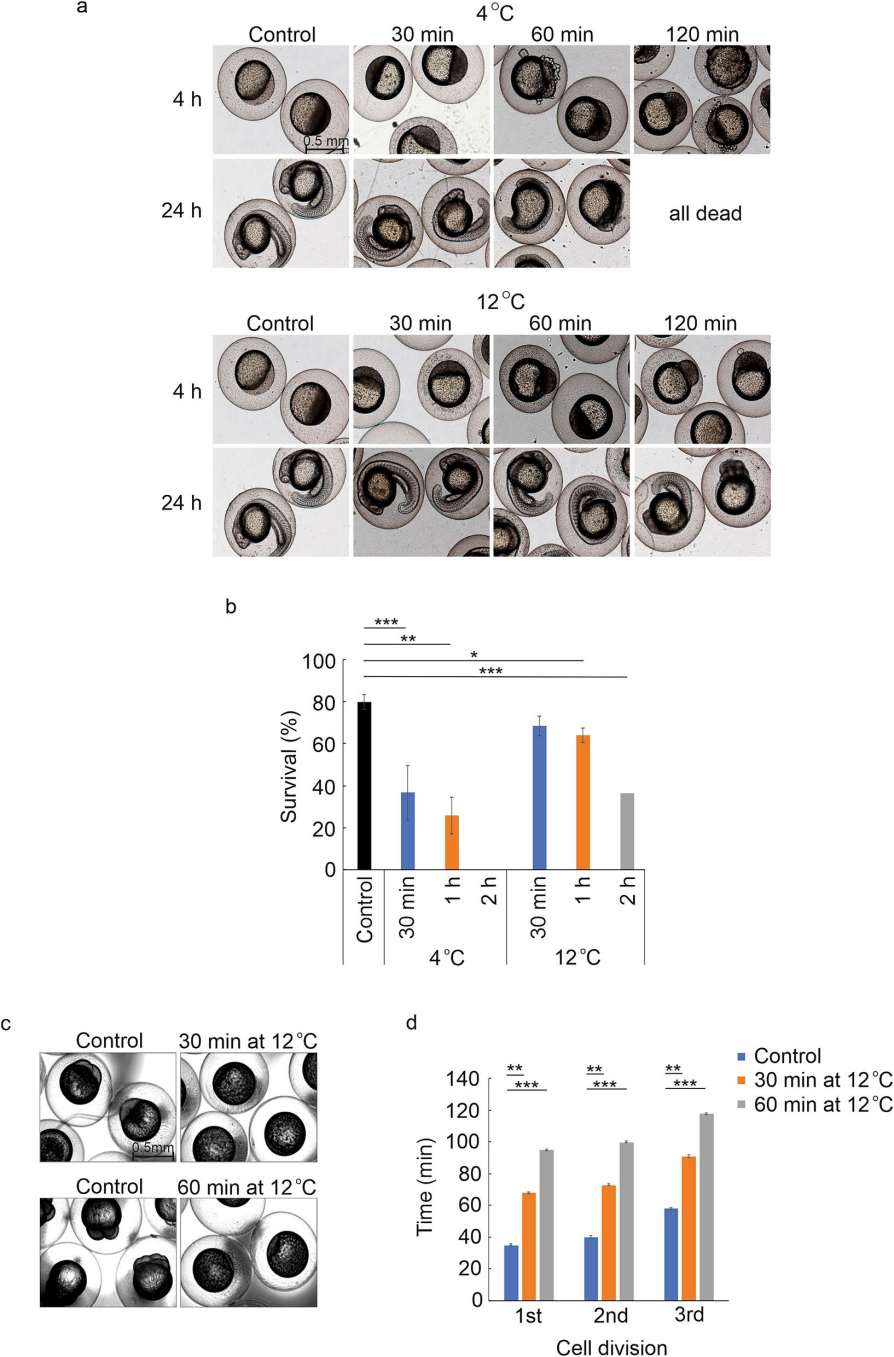
The effect of low temperatures on the early development of zebrafish embryos. (**a**,**b**) 30 min incubation of fertilized eggs in 4 °C causes developmental defects and increased mortality while the environmental temperature of 12 °C is better tolerated causing only moderate defects and mortality after 2 h incubation. Newly fertilized eggs were kept in E3 water at 4 °C or 12 °C for 30 min, 1 h or 2 h after which they were moved to 28 °C. The survival and well-being of the embryos was followed for 24 h and compared to control embryos kept in 28 °C throughout the experiment. Figures represent combined results from 3 independent experiments. n(control) = 353, n(30 min/4 °C) = 278, n(1 h/4 °C) = 335, n(2 h/4 °C) = 247, n(30 min/12 °C) = 384, n(1 h/12 °C) = 396, n(2 h/12 °C) = 323. (**c**) The comparative developmental stages of the control (E3 treated) and the cold treated embryos. Newly fertilized eggs were kept in 12 °C for 30 or 60 min and the development was followed by time-lapse imaging. (**d**) The times for the first, the second and the third cell division in cold treated (12 °C) and control (E3 treated) embryos. Time-lapse experiments were repeated twice. Error bars represent SD, *p < 0.05, **p < 0.01, ***p < 0.001).

Next, we analyzed the effect of the reduced environmental temperature on the early development of zebrafish embryos. We tested the effect of two extreme temperatures (4 °C and 12 °C) and several incubation times on survival rates and well-being of the embryos. Right after laying, eggs were placed in E3 water to 4 °C or 12 °C for 30 min, 1 h or 2 h after which they were moved to 28 °C. Embryos were then checked and imaged at 4 and 24 hpf. Noteworthy, incubation of eggs at 4 °C even for 30 min significantly decreased survival (37%) compared to control group kept in 28 °C throughout the experiment (survival 80%) (p = 0.0004) and after 2 h incubation there were no surviving embryos (Fig. 3a,b). Similarly, incubating embryos in 12 °C for 2 h resulted in significantly lower survival (36%) than in control group (80%) (p = 0.0002) (Fig. 2b). On the other hand, incubation at 12 °C for both 30 min and 1 h was better tolerated by the embryos resulting in survival rates of 69% (p = 0.1) for 30 min and 64% (p = 0.02) for 1 h, compared to the control group with the survival of 80% (Fig. 3a,b). Next, we analyzed whether incubation at 12 °C for 30 min or 1 h can delay the first cell divisions in zebrafish embryos. For this, we collected zygotes immediately after fertilization and incubated them at 12 °C for either 30 min or 1 h. After incubation, the embryos were transferred to the Cell-IQ system where time-lapse imaging took place for 6 h at 28 °C. Corresponding control group embryos were collected and kept at 28 °C for the same incubation time after which they were placed into the Cell-IQ for time-lapse imaging. Comparison of the time-lapse images of embryos with no treatment, 30 min treatment or 1 h treatment revealed a developmental delay in 12 °C treated embryos (Fig. 3c,d, Supplementary Video S1). For the embryos kept at 12 °C for 30 min, the times for the first, the second, and the third cell division were 68, 73, and 91 min respectively, resulting in a 33 min delay in every cell division compared to the control group with the corresponding times of 35, 40, and 58 min (p < 0.01) (Fig. 3d). Consistently, in the embryos kept in cold for 1 h, cell division was delayed by 1 h with exact times for the first, the second and the third cell division being 95, 100, and 118 min (p = 0.0003) (Fig. 3d). In general, the delay in the time of cell divisions equaled with the time of cold treatment. In conclusion, by incubation at 12 °C for 30 min or 1 h, the very first cell divisions in zebrafish embryos can be delayed without causing detrimental effects on the morphology or survival.

### Analysis of CRISPR/Cas9 mutagenesis efficiency in zebrafish embryos incubated at 12 °C after injection

Next, we analyzed how the reduction of temperature to 12 °C affects CRISPR/Cas9 efficiency in zebrafish embryos. First, we analyzed the effect of temperature on nuclease activity at 28 °C and 12 °C in vitro. We carried out the in vitro digestion assay using about 1 kb long exon 3 of *carbonic anhydrase VI* (*ca6*) gene as a template and a *ca6* specific sgRNA described in detail in Uusi-Mäkelä et al.^13^. We observed no difference in in vitro activity of Cas9 enzyme at 28 °C and 12 °C (Supplementary Fig. S2).

**Figure 4:**
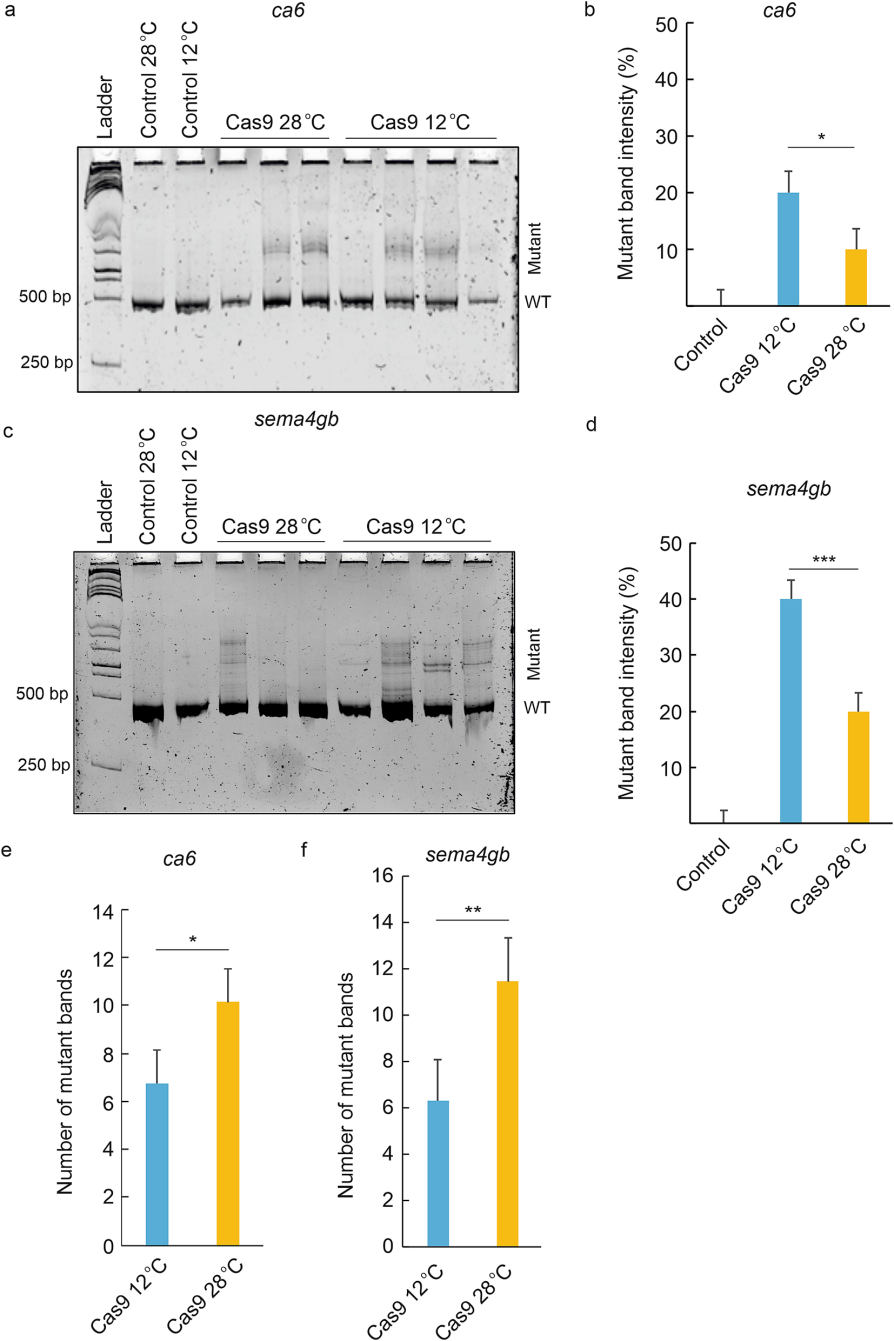
The effect of cold treatment on CRISPR/Cas9 mutagenesis efficiency in zebrafish embryos. Incubating zebrafish embryos in 12 °C immediately after Cas9/sgRNA injections increases the mutagenesis efficiency compared to embryos incubated in 28 °C. (**a**) Representative image of the heteroduplex motility assay (HMA) to analyze the mutagenesis efficiency of Cas9/sgRNA(*ca6)* injected embryos incubated in 12 °C versus 28 °C. The image shows the HMA result for one uninjected (control) embryo incubated in 28 °C, one uninjected embryo incubated in 12 °C, three Cas9/sgRNA(*ca6*) injected individuals incubated in 28 °C (Cas9 28 °C) and four Cas9/sgRNA(*ca6*) injected individuals incubated in 12 °C (Cas9 12 °C). (**b**) Mutant band intensities for control and Cas9/sgRNA(*ca6*) injected embryos. Figure represents combined results from 2 independent experiments, n(Cas9 12 °C) = 76, n(Cas9 28 °C) = 72, n(control 12 °C) = 54, n(control 28 °C) = 50. (**c**) Representative image of the heteroduplex motility assays (HMA) carried out to analyze the mutagenesis efficiency of Cas9/sgRNA(*sema4gb)* injected embryos incubated in 12 °C versus 28 °C. The image shows the HMA result for one uninjected (control) embryo incubated in 28 °C, one uninjected embryo incubated in 12 °C, three Cas9/sgRNA(*sema4gb*) injected individuals incubated in 28 °C (Cas9 28 °C) and four Cas9/sgRNA(*sema4gb*) injected individuals incubated in 12 °C (Cas9 12 °C). (**d**) Mutant band intensities for control and Cas9/sgRNA(*sema4gb*) injected embryos. Figure represents combined results from 2 independent experiments, n(Cas9/12 °C) = 68, n(Cas9/28 °C) = 66, n(control 12 °C) = 52, n(control 28 °C) = 48. (**e**) Number of mutant bands in 12 °C or 28 °C treated, Cas9/sgRNA(*ca6*) injected embryos, after heteroduplex formation, n(Cas9/12 °C) = 54, n(Cas9/28 °C) = 49. (**f**) Number of mutant bands in 12 °C or 28 °C treated, Cas9/sgRNA(*sema4gb*) injected embryos, after heteroduplex formation, n(Cas9/12 °C) = 52, n(Cas9/28 °C) = 47. Error bars represent SD, *p < 0.05, **p < 0.01, ***p < 0.001), *WT *Wild-type band.

To analyze the effect of lowered temperature on the in vivo mutagenesis efficiency, we chose two sgRNAs previously used in Uusi-Mäkelä et al.^13^, a highly efficient *ca6* targeting sgRNA, and a sgRNA with low efficiency, targeting the *sema4gb* (*sema domain, immunoglobulin domain (Ig), transmembrane domain (TM) and short cytoplasmic domain, semaphorin 4 Gb*) gene. In the experiment, embryos were injected with Cas9/sgRNA mixture at room temperature, incubated at 12 °C for 30 min (controls at 28 °C) and moved to 28 °C. For each embryo, the cold exposure started approximately 10 min post fertilization. At 48 hpf, embryos were harvested, and mutated genomic site was analyzed using heteroduplex motility assay^14^. The calculations of the mutagenesis efficiencies were made by comparing the signal from the wt and mutant bands in Cas9/sgRNA injected embryos kept in 12 °C or 28 °C after heteroduplex formation. Importantly, our results showed that cold exposure does not cause significant effect on survival of the injected embryos. When the mutant band intensity of Cas9/sgRNA(*ca6*) injected embryos incubated at 12 °C (20%) was compared to the mutant band intensity of Cas9/sgRNA (*ca6*) injected embryos incubated at 28 °C (10%), we detected a 10% increase (p = 0.01) in the mutagenesis efficiency upon treatment at 12 °C (Fig. 4a,b, Supplementary Fig. S3). Similarly, incubation at 12 °C significantly (p = 0.00004) improved Cas9 mutagenesis efficiency on *sema4gb* resulting in mutant band intensity of 40% compared to 20% for 28 °C incubation (Fig. 4c,d, Supplementary Fig. S3).

As the number of heteroduplex (or mutant) bands after heteroduplex formation can give insight into the level of mosaicism, we also compared the number of these bands in 12 °C or 28 °C treated CRISPR injected embryos and found that that the number of mutant bands was significantly lower for both targeted genes when the embryos were incubated in 12 °C, indicating lower level of mosaicism (Fig. 4e,f). The median number of bands in Cas9/sgRNA(*ca6*) injected embryos incubated in 28 °C was 10.1, and for the embryos treated in 12 °C 6.5 (p < 0.05) Similarly, upon cold treatment the number of mutant bands in Cas9/sgRNA(*sema4gb*) was decreased from 11.5 to 5.3 (p < 0.01). Our data therefore suggests that the incubation of Cas9-sgRNA injected embryos for 30 min at 12 °C provides improved mutagenesis rate and decreased level of mosaicism after CRISPR/Cas9 targeted mutagenesis in zebrafish.

## Discussion

Zebrafish has emerged as a powerful animal model for research and the employment of the CRISPR/Cas9 method has further increased its attractiveness in modelling disease and development. Although regarded as the most powerful tool for genome editing available to date, the relatively low rate of mutagenesis and mosaicism induced by Cas9 and sgRNA injection in zebrafish hampers efficient analyzes of phenotypes and the production of stable mutant lines. To enhance Cas9 driven mutagenesis rate, injection of components to oocytes rather than zygotes followed by in vitro fertilization, has previously been reported by Xie et al.^10^. However, although the method by Xie et al. significantly increased the mutagenesis efficiency, it also resulted in mosaic animals, perhaps due to a short incubation time (30 min) between the injection and in vitro fertilization and the use of Cas9 mRNA instead of a protein^10^. As reported by Xie et al., and by many others, the choice of an appropriate medium is critical for the storage of the oocytes and the success for the in vitro fertilization^12,15–17^. Here, we used the ovarian fluid form Rainbow trout as a novel preservation medium for zebrafish oocytes. As RTOF was able to preserve the oocyte viability for 4 h and fertilizability for 2 h, our study provides an applicable alternative medium for oocyte storage. However, although RTOF preserved the oocyte ability to be fertilized, the obtained survival percentages for the embryos were too low (3% for 2 h incubation) for conducting CRISPR/Cas9 mutagenesis in high-throughput manner. These rather low survival rates even in the control group are not exceptional among the in vitro fertilization studies in zebrafish and mainly result from the varying quality of oocytes and sperm, i.e. parameters which based on the current knowledge are difficult to control^10,15,16^. Our results therefore also highlight the need for an optimized in vitro fertilization protocol for zebrafish. Our results suggest that RTOF provides a practical tool for ex vivo storage of zebrafish oocytes for not only oocyte manipulation but also the study of vertebrate oogenesis and folliculogenesis, and while no ovarian fluid is yet commercially available, this finding may potentially interest both academic and industrial interest groups.

Because our original aim was to create an easily applicable and relatively high throughput method to increase mutagenesis rate in zebrafish, and the observed low fertilization/survival rates required a high number of oocytes to obtain sufficient numbers of mutant embryos for the subsequent analyses, we refocused our attention onto defining ways to delay the first cell division in zebrafish zygotes. We analyzed the effect of RTOF, a “suspended animation-like” state inducer H_2_S^18–20^ and reduced temperature on the rate of the first cell divisions. Previously, it has been shown that H_2_S is able to cause a “suspended animation-like” condition characterized by a decreased metabolic rate and affecting the rate of early development in zebrafish embryos in a concentration dependent manner^18–20^. Similarly, environmental temperature has been shown to have a profound influence on the biological function of ectothermic organisms, such as fish. In zebrafish, the effect of environmental temperature on embryonic development has been studied thoroughly^21,22^. In these studies, it was shown that growing the newly fertilized zebrafish eggs at temperatures lower or higher to their natural conditions (18–33 °C versus 28 °C) changes the developmental rate but also predisposes them to abnormalities^21,22^. In our studies, RTOF and H_2_S did not change the timing of the first three cell divisions in zebrafish embryos. In addition, both induced abnormal development and increased mortality and were thus infeasible for the study, as was the predisposure of embryos to an extreme temperature of 4 °C. Although considerably lower to their natural environment (28 °C), temperature of 12 °C was, on the other hand, well tolerated and resulted in a significant delay of 30–60 min in the first three cell divisions. This condition was therefore tested for our hypothesis, that the developmental delay would give the CRISPR/Cas9 mutagenesis more time to achieve the genetic changes, and thus, result in increased mutagenesis efficiency.

We found that Cas9 and sgRNA injected zygotes showed increased mutagenesis efficiencies at 12 °C compared to the ones incubated at 28 °C throughout the study. With this simple addition in the protocol, we were able to increase the efficiency of CRISPR/Cas9 mutagenesis by about 20%. Notably this increase is likely accompanied with the decrease in the level of genetic mosaicism suggested by decrease in the level of mutant bands at 12 °C versus 28 °C. However, future analysis, using for example Next generation sequencing (NGS) or Tracking of Indels by Decomposition (TIDE) will reveal the exact nature and frequency of mutations^23^ at 12 °C. Similarly, future work will reveal germline transmission rate at 12 °C for different loci. Taken together, our work suggests that incubating CRISPR/Cas9 injected zygotes at 12 °C instead of 28 °C improves mutagenesis rate and reduces mosaicism in zebrafish.

## Methods

### Zebrafish maintenance

Wild-type zebrafish of AB line were obtained from the Tampere Zebrafish laboratory at the Tampere University and the Zebrafish unit of the University of Helsinki. Zebrafish were maintained according to the standard procedure, in a flow-through system with a light/dark cycle of 14 h/10 h. Embryos and larvae under 6 days old were grown in an incubator at 28 °C in embryo medium 3 (E3) (5 mM NaCl, 0.17 mM KCl, 0.33 mM CaCl_2_, 0.33 mM MgSO_4_, 10^–5^% Methylene Blue).

### Ethics statement

All experiments were carried out in accordance with the EU-directive 2010/ 63/EU on the protection of animals used for scientific purposes, with the Finnish Act on the Protection of Animals Used for Scientific or Educational Purposes (497/2013) and the Government Decree on the Protection of Animals Used for Scientific or Educational Purposes (564/2013). Housing and maintenance of zebrafish in the Tampere University was performed under permission of the State Provincial Office of Western Finland (permission ESAVI/10079/04.10.06/2015). In this study, we only used zebrafish prior to their independently feeding larval stages, and thus, no animal permits were required for the experiments. When needed, the embryos and larvae were anesthetized with 0.02% Tricaine (Ethyl 3-aminobenzoate methanesulfonate, pH 7.0) (Sigma-Aldrich, St. Louis, Missouri, USA).

### Rainbow trout ovarian fluid and in vitro fertilization

Ovarian fluid of rainbow trout (*Oncorhynchus mykiss*) was obtained from Savon Taimen Oy Fish Farm (Rautalampi, Finland) and kept frozen until experimental use. Rainbow trout ovarian fluid (RTOF) was melted and centrifuged at 5430 × *g* for 5 min at 4 °C. Supernatant was transferred to a new tube and used in experiments. E3 medium was used as a control solution for oocyte storage. A day before oocyte collection, the females were separated from the males to prevent natural breeding. Next morning, an equal number of males and females were put in the breeding tank, and as soon as the fish started breeding, the females were collected, euthanized with an overdose (0.08%) of Tricaine and placed in a Petri dish on their side. Immediately after euthanasia, the oocytes were harvested by gently pressing the abdomen of the fish resulting in an effortless release of mature oocytes. The desired storage solution (RTOF or E3 water) was immediately added on the oocytes, and the viability of the embryos was monitored for 4 h. Sperm for in vitro fertilization was harvested by the dissection of testes and stored short-term in Ginsburg Fish Ringers solution^24^. The in vitro fertilization was carried out according to the standard procedures^24^.

### Hydrogen sulfide (H_2_S) treatment and cold exposure of embryos

To induce “suspended animation-like” condition in zebrafish embryos, the embryos were incubated in E3 water containing 0, 10, 25, 50, 100 or 200 µM of hydrogen sulfide (H_2_S) solution (Sigma-Aldrich). No further adjustments were made in the solution. To analyze the effect of the cold treatment on embryonic development, fertilized eggs were collected immediately after being laid, placed in E3 water in 4 °C or 12 °C (VWR INCU-Line temperature adjustable incubator) and incubated for 30 min, 1 h or 2 h after which they were directly moved to a 28 °C incubator.

### Selection and production of sgRNAs for CRISPR/Cas9 genome editing

Mutagenesis efficiency of the selected sgRNAs were previously analyzed in Uusi-Mäkelä et al.^13^. From these, we chose one highly efficient guide for *ca6* (*carbonic anhydrase VI*, ENSDARG00000056499) and one low efficiency guide for *sema4gb* [*sema domain, immunoglobulin domain *(*Ig*),* transmembrane domain *(*TM*) and* short cytoplasmic domain*, (*semaphorin*) 4 Gb, ENSDARG00000088143] gene. Briefly, the production of the sgRNAs was carried out by annealing the sgRNA template oligo (Sigma-Aldrich) and the T7 promoter (Sigma-Aldrich) and by in vitro transcription using the MEGAshortscript T7 Transcription Kit (Ambion, CA, USA) (described in more detail in Hruscha & Schmid, 2013^25^. The size and the integrity of the sgRNAs were analyzed with gel electrophoresis (1% agarose in Tris–acetate-EDTA, TAE) and the concentration of the sgRNAs was measured with the Nanodrop 2000 (Thermo Fischer Scientific, Waltham, Massachusetts, USA).

### sgRNA and Cas9 microinjection and the extraction of genomic DNA

The sgRNAs and the Cas9 protein (Protein services, Tampere University) were co-injected into newly fertilized zebrafish eggs with a microinjector (PV830 Pneumatic PicoPump, World Precision Instruments) under a Leica microscope (MXFLIII). Borosilicate injection needles were prepared with a Flaming/Brown micropipette puller (Sutter Instrument) and calibrated by injecting solution into a halocarbon oil droplet to achieve an injection volume of 1 nl. The embryos were aligned on the edge of a microscope slide in a Petri-dish prior to the injection. An injection solution containing 130 ng/μl sgRNA and 250 ng/μl of the Cas9 protein in nuclease-free water was incubated at 37 °C 15 min after which Phenol Red (0.5%, Sigma-Aldrich) was added to visualize injection. The injection solution was introduced to the cell of the 1-cell stage embryo. To analyze the in vivo mutagenesis efficiency, 20 ± 5 injected embryos were collected for DNA extractions at 24 or 48 hpf. For DNA extraction, the embryos were lysed overnight at 55 °C in lysis buffer (10 mM Tris pH 8.2, 10 mM EDTA, 200 mM NaCl, 0.5% SDS). At the end of incubation, lysates were incubated at 98 °C for 20 min and then 10 min at 4 °C. Undigested crude extracts were precipitated by centrifugation at 5000 × *g* for 15 min. 5 µl of the supernatant was used for PCR reaction.

### Heteroduplex motility assay

Targeted loci were amplified from the genomic DNA by PCR using the DreamTaq Hot Start DNA polymerase (Thermo Fischer Scientific) and the primers described in Uusi-Mäkelä et al.^13^ (Table 1) with 35 cycles and the following amplification protocol: (1) Initial denaturation at 95 °C for 5 min, (2) denaturation at 95 °C for 30 s, (3) annealing at Tm (see Table 1) for 30 s, (4) extension at 72 °C for 30 s and (5) final extension at 72 °C for 7 min. 10 μl of the PCR product was subjected to heteroduplex formation according to the protocol represented in Yin et al.^14^. After reaction, the products were run on a 10% polyacrylamide gel. The gel was stained with SYBR Safe (Thermo Fisher Scientific). The mutagenesis efficiency in Cas9/sgRNA injected embryos was quantified by comparing the wild type and mutant band intensities in uninjected and injected embryos. The level of mosaicism was estimated by the number of mutant bands in Cas9/sgRNA injected embryos. Of note, the analysis for the level of mosaicism was done only for the samples with visible bands (and no smear), representing 68–76% of the analyzed embryos.

**Table 1:** Primers and the corresponding PCR products and annealing temperatures used to evaluate the mutagenesis efficiency in zebrafish embryos (PCR-F and PCR-R) or the in vitro activity of Cas9 (IVDA_F and IVDA-R).

Gene	Accession number	Primer sequence (5′–3′)	Product size (bp)	Annealing T (°C)
*ca6*	ENSDARG00000056499	PCR-F: AGCATGCAACACCTTCGGTC	462	57
		PCR-R: ATTTCAGGCATAAGTCCAGC		
		IVDA-F: TAGTCCACGAATGCAACAGC	982	58
		IVDA-R: GGCATGTCTGGCACAAATAG		
*sema4gb*	ENSDARG00000088143	PCR-F: GGACTCACGCCTTCAGAC	381	57
		PCR-R: GCCTTATATCAGCGATGTTAC		
		IVDA-F: ACCCCGCTGTGCTTACATAG	995	60
		IVDA-R: TCACTTTCATTCTGCCCAATC		

### In vitro digestion of DNA with the Cas9-sgRNA complex

To test the in vitro activity of Cas9 on the target genomic sites at different temperatures, equimolar amounts of the endonuclease and sgRNA were pre-incubated 15 min at 37 °C in NEB 3 Buffer (New England Biolabs, Ipswich, Massachusetts, USA) with 1% Bovine serum albumin (Sigma-Aldrich). An approximately 1000 bp site around *sema4gb* and *ca6* target site was amplified using Maxima Hot Start DNA polymerase and the primers described in Uusi-Mäkelä et al.^13^ and in Table 1. Following PCR amplification protocol was used: (1) Initial denaturation at 95 °C for 5 min, (2) denaturation at 95 °C for 30 s, (3) annealing at Tm (see Table 1) for 30 s, (4) extension at 72 °C for 45 s and (5) final extension at 72 °C for 7 min (35 cycles). The resulting products were purified with GeneJET PCR Purification kit (Thermo Fischer Scientific) and used as templates in the digestion assay. The reaction mixture containing 10:10:1 ratio of protein:sgRNA:template was incubated 3 h at desired temperature (12 °C or 28 °C) after which the sample was incubated with 300 U of Proteinase K at 37 °C for 10 min to release Cas9. Proteinase K was inactivated by incubation at 65 °C for 10 min. To analyze the digestion efficiency, samples were run on a 1% agarose TAE gel.

### Time-lapse imaging

For time-lapse imaging and analysis, CM Technologies Cell-IQ Automated Imaging and Analysis System with Nikon Plan Fluor 4×/0.13, WD 16.5 mm (Air) objective were used. Crosses were set a day before and embryos were collected as soon as the fish laid eggs for immediate imaging. Depending on the experiment, the eggs were kept in 6-well plates in E3 medium, RTOF or H_2_S through-out the imaging. For imaging, 1-min cycles were chosen with 8–10 imaging slots. The time of each cell division was manually calculated from the images and compared between the groups.

### Statistical analyses

Data are presented as the mean ± SD. Statistical significance was determined using 1-way ANOVA with a Bonferroni post-test correction, 2-way ANOVA when two variables were present, or the students *t* test comparing control and experimental condition (GraphPad Prism). Differences with a p-value < 0.05 were considered statistically significant.

## Supplementary information

Supplementary Figures.

Supplementary Video 1.

Supplementary Legend.
