# Supplementary material for: Improving CRISPR/Cas9 mutagenesis efficiency by delaying the early development of zebrafish embryos
Source: Sci Rep. 2020 Dec 3;10:21023. doi: 10.1038/s41598-020-77677-9 (PMC7713128; doi:10.1038/s41598-020-77677-9)
Supplement: Supplementary file 1 — Supplementary Figures. [file 41598_2020_77677_MOESM1_ESM.pdf]

Improving CRISPR/Cas9 mutagenesis efficiency by delaying the early development of zebrafish embryos

Terzioglu, M.[1], Saralahti, A.[2], Piippo, H.[2], Rämet M. [2], Andressoo J-O.[1,3*]

[1]Department of Pharmacology, Faculty of Medicine & Helsinki Institute of Life Science, University of Helsinki, Finland
[2]BioMediTech, Faculty of Medicine and Health Technology, Tampere University, Finland
[3]Department of Neurobiology, Care Sciences and Society, Karolinska Institutet, Sweden

* Corresponding author
jaan-olle.andressoo@helsinki.fi
cell: +358 50 358 1213

Supplementary figures

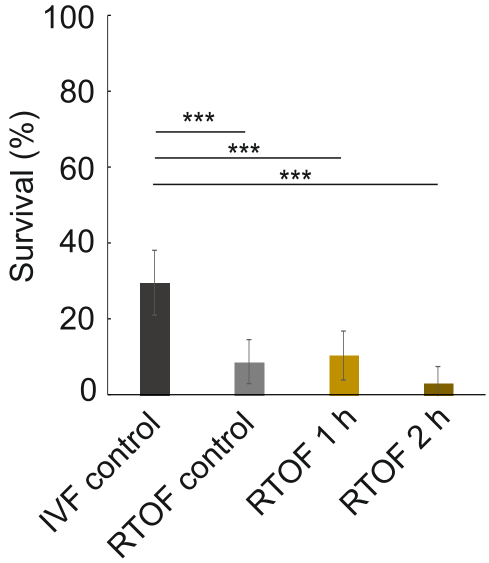

Supplementary Figure S1. Survival percentages 24 h after the *in vitro* fertilization (IVF) of RTOF treated (1 h or 2 h), IVF control (no storage before i*n vitro* fertilization) or RTOF control (30 s storage in RTOF before *in vitro* fertilization). n(IVF control)=567, n(RTOF control)=404, n(RTOF 1 h)=620, n(RTOF 2 h)=731. Error bars represent SD, ***=p0.001.

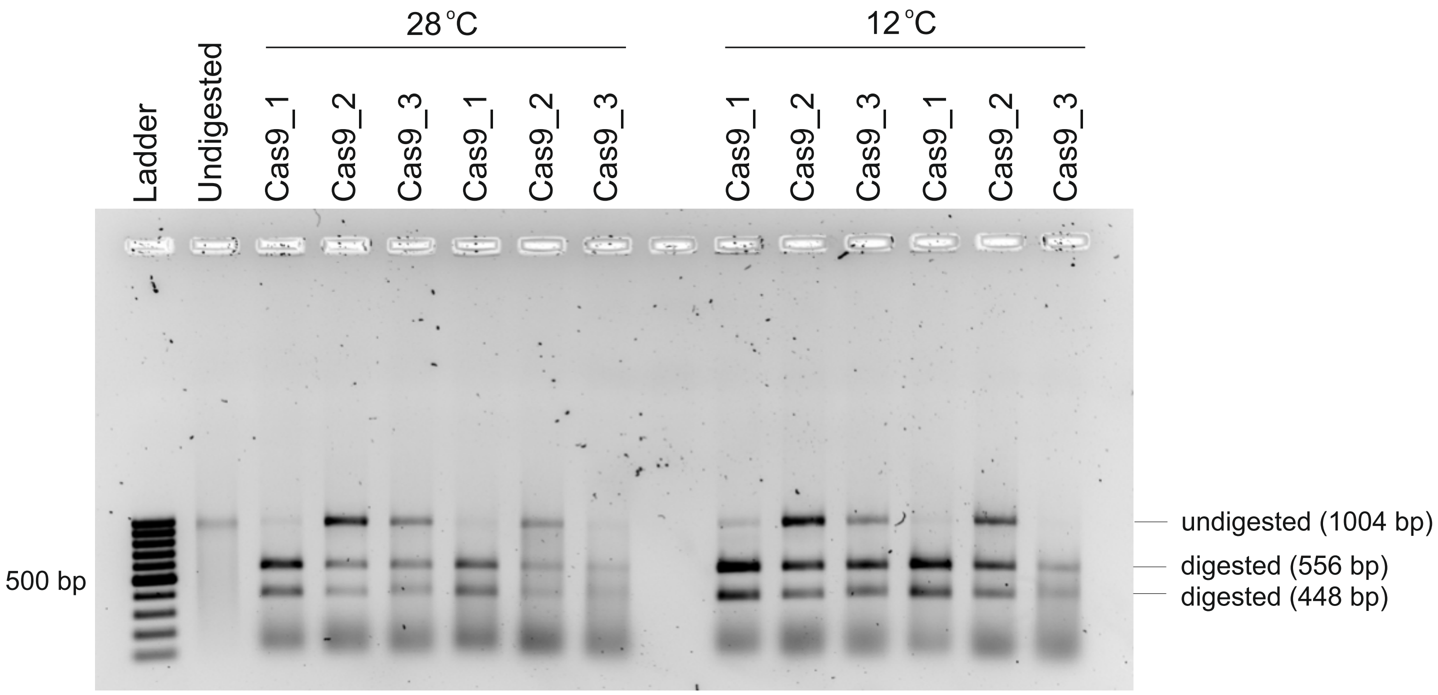

Supplementary Figure S2. *In vitro* Cas9 digestion assay in 28 °C and 12 °C. A PCR product of about 1 kb from the exon 3 of *carbonic anhydrase VI* (*ca6*) gene was *in vitro* digested with Cas9 at 28 °C or 12 °C for 1 h or 2 h and the following products were run on an agarose gel. Three different Cas9 proteins were tested, Cas9_1=protein services, Tampere university (used throughout the study), Cas9_2=commercial Cas9 (ToolGen Inc., Seoul, South Korea), Cas9_3=ExoCas9 (a generous gift from Dr. Daniel S. Wagner, Department of Biosciences, Rice University-Texas, USA [26])

.

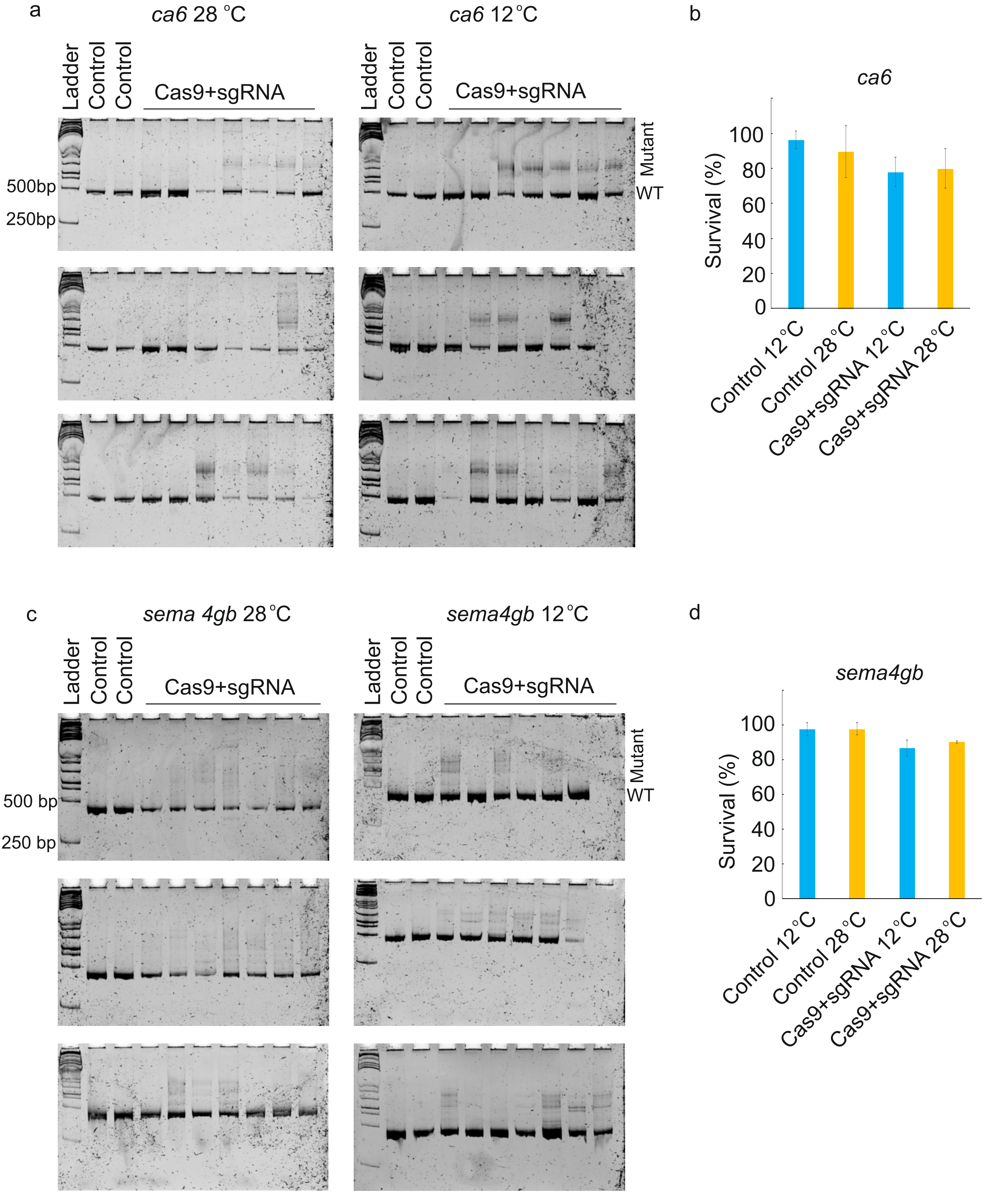

Supplementary Figure S3. Polyacryl amide gel images of the heteroduplex motility assays. (A) Zebrafish eggs were injected with a mixture of gRNA(*ca6*) and Cas9 protein, incubated 30 min in 28 °C or in 12 °C and moved to 28 °C. Mutagenesis efficiency was analyzed with heterodublex motility assay. (B) Survival of Cas9/sgRNA(*ca6*) injected (or control; no injection) embryos. (C) Zebrafish eggs were injected with a mixture of gRNA(*sema4gb*) and Cas9 protein, incubated 30 min in 28 °C or in 12 °C and moved to 28 °C. Mutagenesis efficiency was analyzed with heterodublex motility assay. (D) Survival of Cas9/sgRNA(sema4gb) injected (or control; no injection) embryos. Error bars represent SD. WT=Wild-type band.

Supplementary reference

26. Clements, T. P., Tandon, B., Lintel, H. A., McCarty, J. H., & Wagner, D. S. RICE CRISPR: Rapidly increased cut ends by an exonuclease Cas9 fusion in zebrafish. *Genesis*. **55,** 1–6. (2017).
