# Supplementary material for: Improving CRISPR/Cas9 mutagenesis efficiency by delaying the early development of zebrafish embryos
Source: Sci Rep. 2020 Dec 3;10:21023. doi: 10.1038/s41598-020-77677-9 (PMC7713128; doi:10.1038/s41598-020-77677-9)
Supplement: Supplementary file 3 — Supplementary Legend. [file 41598_2020_77677_MOESM3_ESM.docx]

**Supplementary video S1.** Time lapse imaging of the early development of zebrafish embryos incubated in RTOF versus E3 water, hydrogen sulfide versus E3 water, and in 12 °C (E3 water) versus 28°C (E3 water).
